# Modeling the cognitive processes of accepting clinical decision support

**DOI:** 10.1177/17470218251398419

**Published:** 2025-11-07

**Authors:** Leendert van Maanen, Dominik Bachmann, Talha Özüdoğru, Macy Bouwhuizen, Baptist Liefooghe

**Affiliations:** 1 Experimental Psychology & Helmholtz Institute, Utrecht University, Utrecht, Netherlands; 2 Institute for Logic, Language, and Computation, University of Amsterdam, Netherlands; 3 Social Health & Organizational Psychology, Utrecht University, Utrecht, Netherlands

**Keywords:** Decision making, decision support systems, LBA modeling

## Abstract

People often hesitate to rely on algorithmic advice, even when it is objectively more accurate than human input—a phenomenon known as algorithm aversion. In two experiments, we investigated the cognitive mechanisms underlying this effect in a clinical decision-making context. Participants evaluated X-rays for bone fractures, with each image accompanied by advice purportedly from either an algorithm or a human source. Across experiments, we observed longer response times for algorithmic advice, indicating increased deliberation. Evidence accumulation modeling revealed that participants set higher decision thresholds when evaluating algorithmic advice, reflecting a more cautious decision strategy. This hesitancy, observed when the human advice was attributed to lay participants (Experiment 1), persisted when the human advice was attributed to expert radiologists (Experiment 2). Accumulation rates and prior preferences did not differ across advisor types, suggesting that algorithm aversion stems specifically from increased caution rather than reduced perceived reliability. These findings demonstrate that algorithm aversion manifests as a strategic shift in decision-making and highlight the value of formal cognitive models for understanding trust in artificial intelligence. Our findings advance the theoretical understanding of algorithm aversion by identifying response caution as a core mechanism. More broadly, the results demonstrate how formal models of decision-making can clarify the cognitive architecture of trust in automated systems, offering a foundation for future work on optimizing human–algorithm collaboration.

## Introduction

A common observation in research on *decision support* is that advice from humans and algorithms is trusted and endorsed to a different extent. While some studies report algorithm appreciation, where individuals prefer or trust algorithmic advice over human input (e.g., Araujo et al., 2020; Chacon et al., 2022; Daschner & Obermaier, 2022; Logg et al., 2019), other studies report *algorithm aversion* (Dietvorst et al., 2015), which occurs despite the fact that algorithms often outperform their human counterparts (Filiz et al., 2021; Longoni et al., 2019; Meehl, 1954; Niszczota & Kaszás, 2020; Saha et al., 2024). Also, some research finds no significant differences between the two sources of advice (e.g., Prahl & Van Swol, 2017; Schaap et al., 2024).

Trust in algorithms depends on objective features such as the algorithms’ performance (e.g., reliability, error rate, dependability), automation and transparency (see Daschner & Obermaier, 2022; Glikson & Woolley, 2020; Hancock et al., 2011; Hoff & Bashir, 2015), or the tasks user and system are involved in (e.g., task difficulty or workload, Bogert et al., 2021; Hoff & Bashir, 2015). In addition, trust in algorithms is also driven by the context that shapes psychological responses in users, such as attitudes and beliefs they hold toward algorithms. These attitudes and beliefs emerge at early stages of impression formation (Balas & Pacella, 2015, 2017) and seem independent of the actual performance of an algorithm (e.g., Liefooghe, Min, et al., 2023; Liefooghe, Oliveira, et al., 2023). In medical practice, many diagnoses are already routinely supported by algorithms. So-called Clinical Decision-Support Systems are in place that can help medical practitioners make optimal decisions (Sutton et al., 2020). A recent study by Saha et al. (2024) revealed that an algorithm designed to detect clinically significant prostate cancer outperformed expert radiologists by almost 7%. From a techno-optimistic viewpoint, the expectation is that radiologists who are supported by such algorithms should thus perform on at least the same level (Saha et al., 2024). Consistent evidence, however, points to algorithm aversion in medical contexts (Kaufmann et al., 2023): Both novices and experts are often less willing to accept diagnostic or treatment recommendations from AI, even when algorithmic performance is ostensibly superior (Kaufmann et al., 2023; Longoni et al., 2019; Yun et al., 2021). For example, Filiz et al. (2021) found that participants preferred a human radiologist over an algorithm for interpreting MRI scans, despite being informed of the algorithm’s higher accuracy. This aversion is particularly pronounced in medical contexts involving serious outcomes or moral considerations, where decisions are seen as highly personal or ethically significant (Bigman & Gray, 2018).

As our review of the literature indicates, there has been a lot of research on how features of algorithms, users, and decisions influence algorithmic trust (e.g., Castelo et al., 2019; Glikson & Woolley, 2020; Mahmud et al., 2022). Less attention has been given to the nature of the cognitive processes underlying advice taking from algorithmic and human advice (but see Kamaraj & Lee, 2022; Strickland et al., 2021). Here, we contend that it will be easier to identify what predicts advice taking once we understand the mechanisms by which humans accept or reject advice. Eventually, such an information-processing approach (Proctor & Vu, 2010) could be used to adjust the context in which algorithms are deployed (see also Liefooghe & Van Maanen, 2023).

**Figure 1.**
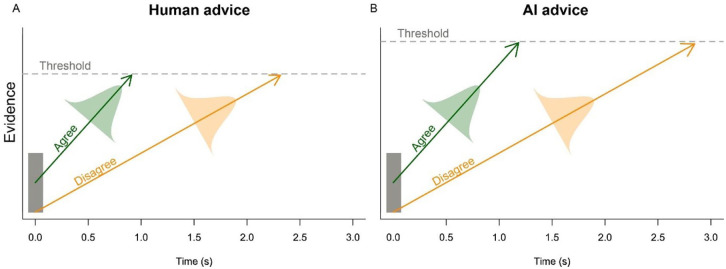
Example linear ballistic accumulator process. (a) A lower threshold is hypothesized for human advice. (b) A higher threshold is hypothesized for AI advice.

One important class of models to study decision-making and, by extension, decision-making in view of algorithmic advice are Evidence Accumulation Models (EAMs, Boag et al., 2024; Mulder et al., 2014; Ratcliff et al., 2016). These models predict both a distribution of choices (e.g., how often people choose “agree” or “disagree” in a binary choice task) and a distribution of Reaction Times (RT; how long it takes people to make these choices), given a set of cognitive processes that are assumed to underly people’s decision-making. First (and foremost), EAMs assume that people (gradually) accumulate information as evidence in favor of different competing choice options. In the present case, such evidence will either lead to the endorsement of algorithmic advice or its dismissal (Figure 1a). The average speed with which evidence is accumulated is typically referred to as the *accumulation rate* (or *drift rate*, the arrows in Figure 1a). It is typically assumed that when all evidence for a particular choice option is consistent, accumulation will be faster (here represented by a steeper arrow). In the case of advice taking, that would mean that advice that is consistent with one’s own beliefs leads to a faster accumulation of evidence.

Evidence accumulation continues until enough evidence has been collected to make a choice—that is, until the amount of accumulated evidence exceeds a *threshold*. How high a threshold is set has a large influence on decision behavior, as a higher threshold leads to slower choices (Heitz, 2014; Katsimpokis et al., 2020, Figure 1b). At the same time, a higher threshold also yields more accurate choices, because more misleading information would need to be accumulated to cross such a threshold (and consequently make an incorrect choice). A third process that is incorporated in EAMs is a prior preference for particular outcomes, modeled by the *starting point* of evidence accumulation. That is, do individuals a priori weigh each choice option equally, or do they display a preference for one option over the other? Such a process is represented by evidence accumulation beginning closer to the threshold of one choice option (relative to the thresholds of other options, Figure 1), making it a priori more likely that this option is chosen (Mulder et al., 2012). Together, these three mechanisms explain the observed distributions of responses and response times. By estimating the set of parameter values that best explain the observed distributions, EAMs can be used to infer the cognitive processes involved in decision-making tasks (Boag et al., 2024), including those involving decision support systems (Strickland et al., 2021, 2023).

In the present study, we used the EAM framework to investigate the cognitive signature of accepting advice from either humans or algorithms. To this end, participants performed a diagnostic task in which X-ray images were presented. Each X-ray either contained a fractured bone or not and was accompanied with advice stemming (purportedly) from an algorithm or a human. The advice converged with the X-ray on the majority of trials, and participants were tasked to endorse the advice or not depending on its accuracy. This way, we framed the cognitive processes involved in advice taking as a binary decision between accepting or rejecting the presented advice. In our design, algorithm aversion or appreciation is measured by comparing the proportion of agreement with human and algorithmic advice. Because faster responses can indicate more certainty in one’s decision (Rahnev et al., 2020), decision times can also be used to understand the mechanisms behind endorsement or rejection of advice.

Based on previous research on algorithm aversion in the context of medical decision making (Kaufmann et al., 2023; Longoni et al., 2019; Yun et al., 2021), we hypothesized that human advice is preferred over algorithm advice. Accordingly, the proportion of agreement with the advice was predicted to be higher for human advisors than for algorithm advisors. Under the assumption that decision times are also sensitive to the nature of the advisor, algorithmic support was also expected to lead to longer decision times compared to human support. Moreover, in line with the extant literature (Appelganc et al., 2022; Filiz et al., 2021; Longoni et al., 2019; Yeomans et al., 2019; Yun et al., 2021), we hypothesized that algorithm aversion is based on a predisposition to distrust algorithms. In terms of the EAM framework, we thus predicted that algorithm aversion is mainly driven by a tendency to set a higher decision threshold for algorithmic advice than for human advice, reflecting a relative hesitancy to accept algorithmic advice (cf. Figure 1b with a).

We present two experiments in which we test the aforementioned predictions. In Experiment 1, we observed longer decision times for algorithmic support compared to human support. The EAMs, furthermore, indicated that larger response thresholds were used for algorithm advice than for human advice. However, the rates at which participants accepted advice did not differ based on whether the advice stemmed from humans or algorithms. In Experiment 2, we aimed to increase algorithm aversion by telling participants that the human advisors were expert radiologists (compared to naïve participants in Experiment 1). The key findings of Experiment 1 were replicated. Both experiments were approved by the local ethics committee at Utrecht University (23-0469).

## Experiment 1

### Methods

#### Participants

Experiment 1 was conducted on Prolific (www.prolific.com). Forty-seven individuals (27 female, mean age 34 years, SD 10 years) who self-reported being fluent in English participated in Experiment 1 in exchange for a standard monetary compensation. One participant was excluded for not following instructions, leaving a sample of 46 participants. All participants provided written informed consent prior to participation.

#### Materials

**Figure 2.**
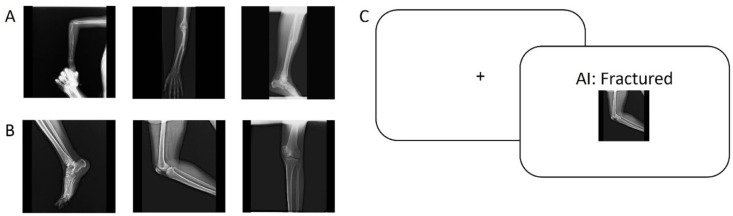
Experimental paradigm. (a) Example fractured X-rays. (b) Example non-fractured X-rays. (c) Experimental Design. *Note.* Advisor (AI vs. human) varied by block, congruency varied by trial.

The stimulus materials were X-ray images taken from FracAtlas (Abedeen et al., 2023). From this data set, we extracted images with and without fractures. We included images from hands, arms, legs, and feet that were free of additional objects (e.g., caskets or jewelry) and annotations. Moreover, the X-ray images always displayed one limb only. Two independent raters classified the difficulty of detecting a fracture on a five-point scale (1: *hard*, 5: *easy*). We ordered the images based on the average rating and selected the 98 easiest images determined this way (a third rater acted as a tiebreaker). Additionally, we selected 98 images that did not show fractures. We made sure that the number of images displaying arms and legs was the same for fractures and non-fractures. The selected images were rotated such that they all had the same orientation, and put on the same black background to form a 450 × 450 pixel square (Figure 2a and b). Depending on the screen type of the participant, and assuming an 80 cm viewing distance, this subtends to a viewing angle between 5° and 8°.

#### Experimental design

On each trial, after a fixation cross (1,000 ms), participants were shown a stimulus on the center of the screen, above which it was shown who the advice was from, and what it said (Figure 2c). There were four options. In a blocked manner, participants were told that the advice came from an algorithm or from other participants in the experiment (we refer to this factor as Advisor). Specifically, participants were informed that the advice was based on the majority advice from another group of individuals in a similar task. In the first case, participants would see “AI: Fractured” if the advice was that the image contained a broken bone, and “AI: Not fractured” if the advice was that the image did not contain a broken bone. In the second case, participants would see “Other participants: Fractured” and “Other participants: Not fractured”, respectively. Participants indicated with a button press whether they “Agreed” (F key) or “Disagreed” (J key) with the advice. Participants received a message indicating that they should respond faster, after a random and trial-by-trial-varying amount of time had passed.^1^ This procedure ensured that participants responded quickly, while avoiding that they learn a specific response deadline (which could influence their behavior; Katsimpokis et al., 2020; Miletić & Van Maanen, 2019).

#### Procedure

The experiment was advertised on Prolific as a data-labeling job to validate AI in image recognition. After signing an informed consent, the actual experiment started. Participants were told that the validation study focused on how accuracy of an image-classification algorithm compared to accuracy of human judgments. After this general instruction and three practice trials to familiarize participants with the experimental setup, participants either started with the “AI advice” block or the “Other participants advice” block (counter-balanced across participants). Each block consisted of 196 trials, half of which displayed Fractured and Non-Fractured stimuli, respectively. All images appeared once in each block. To ensure that participants perceived advice to be trustworthy, the advice was accurate on approximately 65% of the trials (Due to a coding error, the advice was correct on 67% of the trials for human advice, and 64% of the time for AI advice). We refer to this factor as the Congruency of the advice with the stimulus. After the main experiment, participants answered a shortened version of the General Attitudes toward Artificial Intelligence Scale (Schepman & Rodway, 2020). Participants were asked to indicate their agreement with 11 statements concerning people's attitude toward AI on a 5-point Likert scale. The questionnaire results are not reported here (but see OSF for the raw data). The total duration of Experiment 1, including instructions and questionnaire, was 26 min.

### Data analysis

#### Descriptive statistics

We first analyzed the RTs and choices separately, using mixed-effects regression models (Baayen et al., 2008). For these analyses, we excluded trials that were more than 2 SD from each participant’s mean RT. We first defined the most complex model. For the RT analysis, this is a model where RT is modeled as a function of Advisor (human or AI), Congruency (correct or incorrect advice), and Fractured (Yes, No), including all interactions. As random intercepts, we included participants and items. After fitting that model to the data, we stepwise deleted the factors that explained the least amount of variance, and compared those models using BIC. The full analysis script can be found on https://osf.io/3jz4u.

For the choices, we followed the same basic analysis plan, but instead modeled the probability of agreeing with the advice as a logistic function of these same factors.

#### Linear Ballistic Accumulation

We fit the Linear Ballistic Accumulator (LBA) model (Brown & Heathcote, 2008) to the data of Experiment 1. To this end, we adapted LBA model code (Nicenboim, 2018) that was written in Stan (Carpenter et al., 2017). Specifically, we adjusted the code in such a way that not only the accumulation rates could differ between options, as is standard, but also the distance to threshold and the non-decision time. This change allowed us to implement the four different model specifications explained below. The models differed in which parameters we estimated to be equal or different across conditions and choices, influencing the models’ complexity. Using a leave-one-out information criterion (Vehtari et al., 2017), the models were compared on their balance between goodness-of-fit and complexity (i.e., degrees of freedom), as more complex models tend to fit data better but at the risk of overfitting (Pitt & Myung, 2002).

In total, eight models were fit to the data of Experiment 1 (Table 1). Going from Model 1 to 4, the models become less complex, in that more parameters are constrained to be the same across specific conditions. Model 1 was the most complex model. In this model, we allowed both accumulation rate and threshold distance to vary across Advisor, Congruency, and Choice (Agree/Disagree). Model 2 was similar to Model 1, except that we did not allow accumulation rate to vary across Advisor levels. Model 3 was a further simplification to Model 2, where we allowed the distance to threshold to vary between Advisors, but not between Congruency conditions. This way, we tested the hypothesis that participants may not have been biased against algorithmic advice, but rather respond in a more cautious way, leading to an overall larger distance to threshold. Model 4, finally, simplified Model 3 by testing the hypothesis that distance to threshold only depended on Advisor, not Choice. For each model, we fit an *a* and a *b* version. In the *a* version, the non-decision time parameter could vary between the two choices (Agree/Disagree), reflecting that participants might have a different motor process for a left or right button press. In the *b* version of the models, the non-decision time was constrained to be the same value, reflecting the possibility that there are no differences between left and right button presses.

**Table 1.** Model comparison for Experiment 1.

Model	Congruency	Advice	Choice	LOOIC
1a	*v, d*	*v, d*	*d, t_0_*	43,400
1b	*v, d*	*v, d*	*d*	43,427
2a	*v, d*	*d*	*d, t__0__*	**43,330**
2b	*v, d*	*d*	*d*	43,344
3a	*V*	*d*	*d, t__0__*	43,660
3b	*V*	*d*	*d*	43,764
4a	*V*	*d*	*t__0__*	43,718
4b	*V*	*d*		43,556

*Note.* v = accumulation rate; d = distance to threshold; t_0_ = non-decision time; LOOIC = Leave-one-out information criterion.

Bold denotes the model that best balances goodness-of-fit and model complexity.

Besides the aforementioned parameters (accumulation rate, threshold distance, non-decision time), we additionally estimated a starting point variability parameter in each of our models, as is customary for LBA models. Starting-point variability reflects the variability between the starting point of accumulation and the threshold (i.e., the threshold distance). This parameter was considered to be the same in all conditions. The variability across accumulation rates was fixed at 1 such that the model becomes identifiable (Van Maanen & Miletić, 2021). We fit the model on the second scale (Tran et al., 2021).

We assumed that participants’ individual parameters are not independent, and hence estimated both parameters that described the overall distribution of model parameter values at the group level (hyperparameters, e.g., mean and SD) as well as participants’ individual level parameters (e.g., participant 1’s non-decision time). Inferences are based on the group-level estimated posterior distributions. Specifically, we computed what proportion of the posterior distribution for one condition exceeded the posterior distribution of the other condition. This essentially quantifies the probability that one parameter estimate is higher than another. If this proportion is 50%, the distributions cannot be distinguished, and each parameter estimate is equally likely to be the highest. If this proportion is 99%, the probability that one parameter estimate is higher is 99%.

We fit the models using RStan (Carpenter et al., 2017), using four chains and 1000 iterations, of which 300 were burn-in. As priors, we used the same priors as Nicenboim (2018). Additionally, we made sure that the sampler started in a region of the parameter space that was identified. All models fully converged with $\hat{R}$ =1.

### Results

#### Descriptive statistics

The best model, according to BIC, included all main effects on RT, but no interactions. Relative to the Fractured stimuli, Non-fractured stimuli were slower by 149 ms (*t* = 7.25; *p* < .001). Additionally, there was a Congruency effect, with Congruent advice being 125 ms faster than Incongruent advice (*t* = 6.18, *p* < .001). Importantly, there was an effect of Advisor with responses to human advice being 90 ms faster than responses to AI advice (*t* = 10.39, *p* < .001).

In terms of agreement with the advice, the best model according to BIC was a model that did not include Advisor as a main effect, but did include Fracture and Congruency, as well as their interaction. Participants agreed substantially less on Incongruent trials than on Congruent trials (26% vs. 82%, *z* = 17.57, *p* < .001). Participants also agreed 8% more on Congruent Fractured stimuli as compared to Congruent Non-Fractured stimuli (*z* = 4.48, *p* < .001). However, the proportion agreement was lower for Incongruent Non-fractures as compared to Incongruent Fractures (13%, *z* = 6.31, *p* < .001).

#### Linear ballistic accumulation

**Figure 3.**
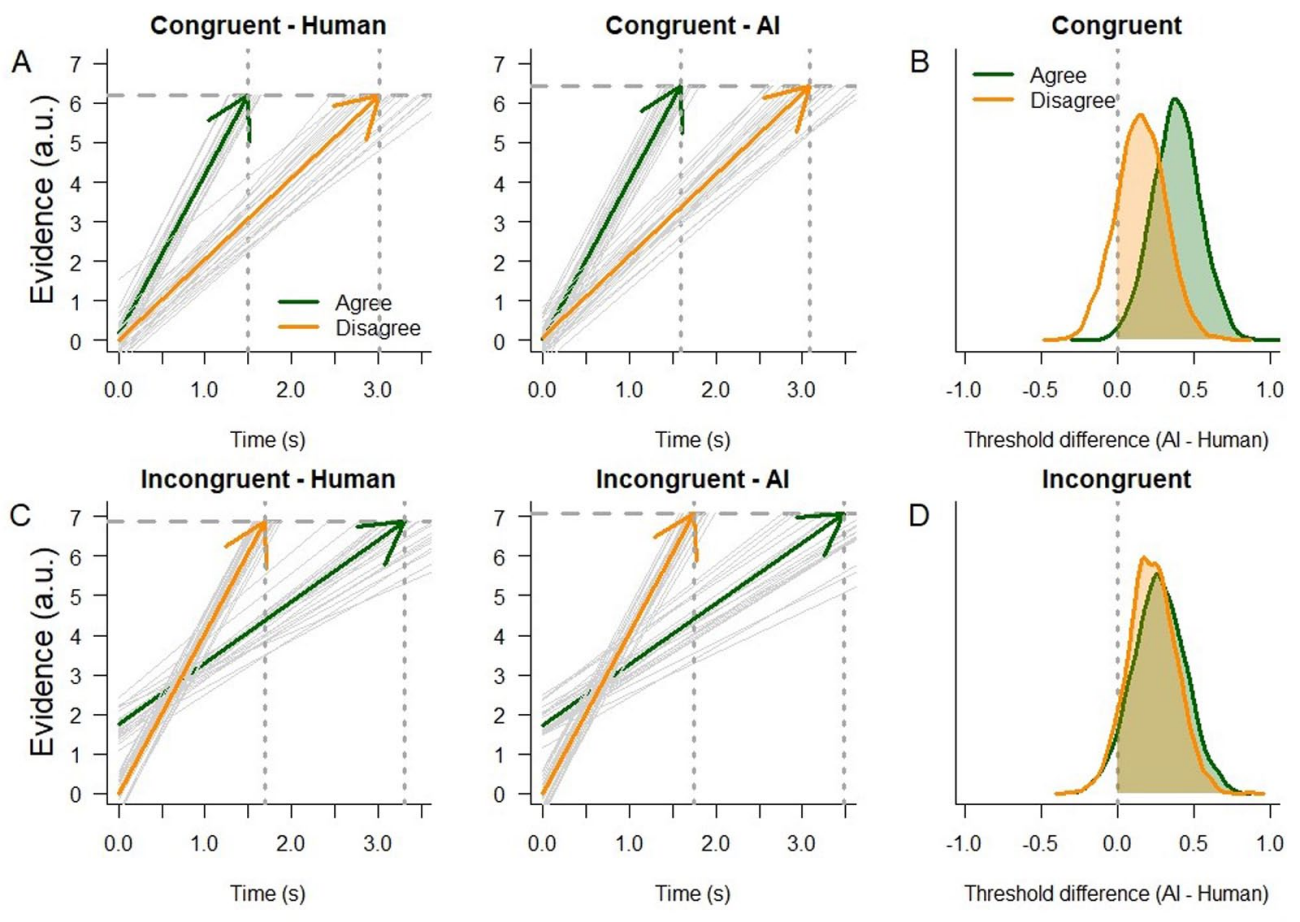
Results of Experiment 1 support the hypothesis that AI advice requires a higher threshold. (a) Experiment 1 shows higher accumulation rates for agree decisions than for disagree decisions on congruent trials, for both Human (left) and AI (right) advice. Thick lines represent the mean a posteriori estimate, the thin grey lines are 25 randomly sampled posterior estimates, to illustrate uncertainty in the estimates. Vertical dashed lines indicate the predicted mean RTs. The threshold is estimated to be slightly higher for AI advice (right) than for Human advice (left). (b) Posterior distributions for differences in threshold between AI and Human advice. Since most posterior mass is positive, AI thresholds are systematically higher. (c) Same as (a), but for Incongruent trials. In this condition, accumulation rates for disagree decisions exceed accumulation rates for agree decisions. Additionally, there is a prior preference to agree, visible as a higher starting point of accumulation. (d) Same as (b), but for Incongruent trials.

Model 2a best balanced model complexity and goodness-of-fit to the data of Experiment 1 (lowest Leave-one-out information criterion (LOOIC), Table 1, the fit of the model is shown in the online Supplemental Material A). This model is presented in Figure 3. Figure 3a illustrates the accumulators for the Agree and Disagree choices on Congruent trials, split by Human or AI advice. Accumulation rates for the Agree choice are consistently larger than for the Disagree choice. Note the slightly higher threshold for AI advice. Comparing the posterior distributions of the threshold estimates shows that, indeed for AI advice, the threshold is consistently larger (Figure 3b). For Agree choices, this was the case for 99% of the samples; For Disagree choices, this was the case for 82%.

On Incongruent trials, the opposite pattern of accumulation is visible (Figure 3c). In this case, the accumulation rate of the Disagree choice is larger than the accumulation rate of the Agree choice. Again, the distance to threshold is higher for AI advice than for Human advice. 94% of the samples of the posterior distribution of the AI advice threshold for the Agree choice were larger than the Human advice threshold (93% for the Disagree choice, Figure 3d).

Additionally, participants exhibited a prior preference for agreeing rather than disagreeing (Probability that the Agree decisions have a lower distance to threshold than the Disagree decisions is 100%). Not shown are the posterior distributions for the non-decision time of Model 2a. These were comparable, with the proportion of samples from the Agree choice that is higher than the Disagree choice at 68%. Table 2 displays mean estimates and credible intervals for each parameter of Model 2a.

**Table 2.** Parameter estimates for Model 2a, Experiment 1.

Parameter	Condition	Mean	95%CI
*v*	Congruent, agree	4.01	[3.68, 4.34]
*v*	Congruent, disagree	2.06	[1.72, 2.40]
*v*	Incongruent, agree	1.54	[1.15, 1.93]
*v*	Incongruent, disagree	4.05	[3.73, 4.37]
*d*	Human, congruent, agree	3.51	[3.09, 3.93]
*d*	Human, congruent, disagree	3.68	[3.31, 4.05]
*d*	Human, incongruent, agree	2.58	[2.21, 2.95]
*d*	Human, incongruent, disagree	4.43	[4.02, 4.84]
*d*	AI, congruent, agree	3.90	[3.48, 4.32]
*d*	AI, congruent, disagree	3.83	[3.47, 4.19]
*d*	AI, incongruent, agree	2.84	[2.47, 3.21]
*d*	AI, incongruent, disagree	4.57	[4.16, 4.98]
*t_0_*	Agree	0.16	[0.02, 0.31]
*t_0_*	Disagree	0.11	[0.01, 0.26]
*A*	—	5.04	[3.73, 6.08]

*Note*. v = accumulation rate; d = distance to threshold; t_0_ = non-decision time; A = Starting-point variability; CI = credible interval.

### Discussion

The results of Experiment 1 offer evidence for algorithm aversion. Participants are slower to accept algorithmic than human advice. However, this effect is not observed in the proportion of agreement. When jointly analyzing the RTs and choices together with EAMs, we observe a higher threshold for decisions related to algorithmic advice than for decisions related to human advice. Participants thus require more evidence before accepting or rejecting algorithmic support.

We also observed a difference in threshold distance between agree and disagree decisions. This seems to reflect a bias for agreeing with advice. In particular, a shorter distance, displayed in Figure 1 as a higher start point of accumulation, means that participants displayed a prior preference for agreeing rather than disagreeing. Given the higher probability that the advice is congruent, a higher prior probability to agree with advice is to be expected.

For congruent trials, accumulation rate for Agree choices was higher than for Disagree choices. For incongruent trials, we observed the opposite pattern. This indicates a successful operationalization of our paradigm: Participants apparently extracted the correct information from the stimulus on the majority of trials. We did not find evidence for a difference in accumulation rates between human and AI advice, as models that included that factor were not preferred by the model comparison metric.

The results of Experiment 1 only in part confirmed our predictions. Decisions were made more cautiously when the algorithm advice was presented, but this effect was relatively weak. In addition, the proportion of agreement did not differ between both types of support. For this reason, we decided to replicate Experiment 1.

## Experiment 2

In Experiment 1, participants were told that human advice was based on the responses of other participants. Such a reference group may not be considered to have sufficient expertise in the X-ray task at hand, perhaps mitigating the difference in perception between human and algorithm advice. Accordingly, in Experiment 2, we told participants that the human advice was based on the expertise of trained radiologists. We reasoned that this would express a relatively high level of expertise in the task. Consequently, we expected algorithm aversion to be stronger, driven by a higher appreciation of human advice.

### Methods

Experiment 2 was conducted on Prolific. Forty-eight individuals (19 female, mean age 34 years, SD 9 years) who self-reported being fluent in English participated in exchange for a standard monetary compensation. All participants provided written informed consent prior to participation. Two participants were excluded for not following instructions, leaving a sample of 46. The design of Experiment 2 was identical to Experiment 1 with the exception of the block manipulation. In Experiment 2, participants were told that the human advice was based on a group of trained radiologists. Consequently, the cue that was presented above the stimuli read “Radiologists: Fractured” or “Radiologists: Not Fractured” in case of the Human advice block, and “AI: Fractured,” “AI: Not Fractured” in case of the AI advice block. The advice was correct on 60% of trials, evenly distributed over the cells of the design. During the instruction phases, the expertise of the individuals in the Human block was emphasized by replacing mentions of “other participants” with “trained radiologists.” The median duration of Experiment 2 was 27 min. The same statistical analyses were performed as in Experiment 1. The data and analyses can be found on https://osf.io/3jz4u.

### Results

#### Descriptive statistics

The best model, according to BIC, included only the effect of Advisor on RT, but no other main effects or interactions. Participants responded 47 ms faster to human advice than to AI advice (*t* = 4.74, *p* < .001).

In terms of agreement with the advice, a model with all main effects, plus the interactions Advisor × Fractured and Congruency × Fractured was preferred. Participants agreed more on Congruent trials than on Incongruent trials (Congruent stimuli 84% agreement, Incongruent stimuli 22%, *z* = 14.70, *p* < .001). Participants also agreed more on Non-Fractured Congruent stimuli than on Fractured Congruent stimuli (88% vs. 84%, *z* = 2.02, *p* = .043). For Incongruent stimuli, this effect was reversed (Fractured: 22%; Non-fractured: 10%, *z* = 4.18, *p* < .001). Finally, while for Fractured stimuli, there was no significant difference between the proportion agreement for Human and AI advice, for Non-fractured Congruent stimuli, participants agreed more with Human advice than with AI advice (92% vs. 88%, *z* = 3.71, *p* < .001).

#### Linear Ballistic accumulation

**Figure 4.**
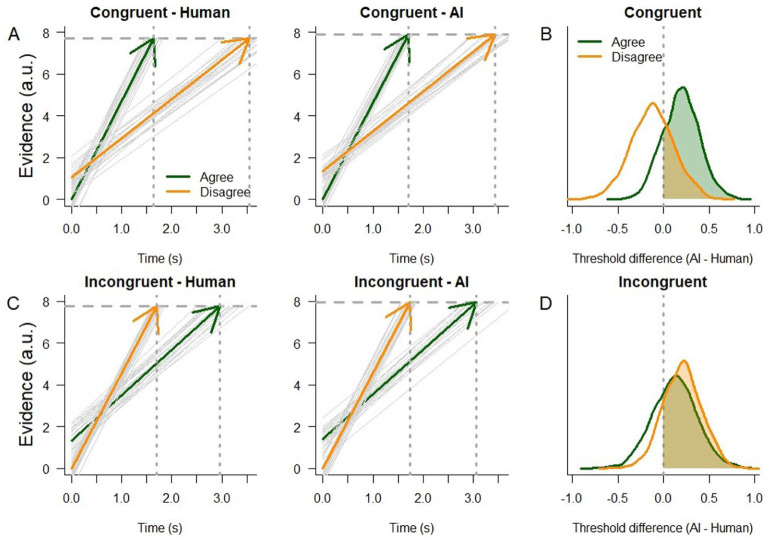
The results of Experiment 2 replicate Experiment 1. (a) Experiment 2 shows higher accumulation rates for agree decisions than for disagree decisions on congruent trials, for both Human (left) and AI (right) advice. Thick lines represent the mean a posteriori estimate, the thin grey lines are 25 randomly sampled posterior estimates, to illustrate uncertainty in the estimates. Vertical dashed lines indicate the predicted mean RTs. The threshold is estimated to be slightly higher for AI advice (right) than for Human advice (left). (b). Posterior distributions for differences in threshold between AI and Human advice. Since most posterior mass is positive, AI thresholds are systematically higher. (c) Same as (a), but for Incongruent trials. In this condition, accumulation rates for disagree decisions exceed accumulation rates for agree decisions. Additionally, there is a prior preference to agree, visible as a higher starting point of accumulation. (d). Same as (b), but for Incongruent trials.

In contrast to Experiment 1, the model that best performed according to LOOIC was Model 1a, the most complex model (Table 3, the fit of the model is shown in the online Supplemental Material B). However, inspecting the estimated parameters reveals similar patterns as in Experiment 1. On Congruent trials, accumulation rates were higher for agree choices than for disagree choices, for both Human and AI advice (100%, Figure 4a). Again, this pattern was reversed on Incongruent trials (Figure 4c). In agreement with Experiment 1, accumulation rates were the same for AI and Human advice (51% probability that the AI accumulation rate is higher than human accumulation rate).

**Table 3.** Model comparison for Experiment 2.

Model	Congruency	Advice	Choice	LOOIC
1a	*v, d*	*v, d*	*d, t_0_*	**45,223**
1b	*v, d*	*v, d*	*d*	45,236
2a	*v, d*	*d*	*d, t_0_*	45,370
2b	*v, d*	*d*	*d*	45,355
3a	*v*	*d*	*d, t_0_*	45,761
3b	*v*	*d*	*d*	45,802
4a	*v*	*d*	*t_0_*	45,870
4b	*v*	*d*		45,964

*Note*. v = accumulation rate; d = distance to threshold; t_0_ = non-decision time; LOOIC = Leave-one-out information criterion.

Bold denotes the model that best balances goodness-of-fit and model complexity.

Importantly, the threshold distance was generally higher for AI advice than for human advice both on Congruent trials (82% for Agree choices, but 31% for Disagree choices, Figure 4b) and on Incongruent trials (70% for Agree and 83% for Disagree, Figure 4d). Interestingly, when participants disagreed with advice on Congruent trials, there was no difference in threshold, or perhaps even a lower threshold for AI advice than for Human advice (31% for Disagree choices).

The probability that non-decision times for Agree are higher than for Disagree is 80%. Table 4 displays mean estimates and credible intervals for each parameter of Model 1a.

**Table 4.** Parameter estimates for Model 1a, Experiment 2.

Parameter	Condition	Mean	95%CI
*v*	Human, congruent, agree	4.70	[4.40, 5.00]
*v*	Human, congruent, disagree	1.97	[1.70, 2.24]
*v*	Human, incongruent, agree	2.17	[1.89, 2.45]
*v*	Human, incongruent, disagree	4.55	[4.25, 4.85]
*v*	AI, congruent, agree	4.64	[4.35, 4.93]
*v*	AI, congruent, disagree	1.90	[1.62, 2.18]
*v*	AI, incongruent, agree	2.14	[1.87, 2.41]
*v*	AI, incongruent, disagree	4.60	[4.30, 4.90]
*d*	Human, congruent, agree	4.18	[3.61, 4.75]
*d*	Human, congruent, disagree	3.13	[2.63, 3.63]
*d*	Human, incongruent, agree	2.90	[2.41, 3.39]
*d*	Human, incongruent, disagree	4.23	[3.61, 4.85]
*d*	AI, congruent, agree	4.37	[3.80, 4.94]
*d*	AI, congruent, disagree	3.01	[2.50, 3.52]
*d*	AI, incongruent, agree	3.03	[2.56, 3.50]
*d*	AI, incongruent, disagree	4.43	[2.82, 5.04]
*t0*	Agree	0.18	[0.05, 0.33]
*t_0_*	Disagree	0.12	[0.02, 0.30]
*A*	—	7.06	[5.81, 8.31]

*Note*. v = accumulation rate; d = distance to threshold; t_0_ = non-decision time; A = Starting-point variability; CI = credible interval.

### Discussion

Experiment 2 replicated the key findings of Experiment 1. Both in terms of RT as in terms of the EAMs, we found evidence for algorithm aversion. Decision thresholds were set differently for algorithmic support compared to human support. In contrast, evidence accumulation rates did not differ, nor did the difference in starting points. Finally, we observed that participants agreed more with human advice than with algorithmic advice, at least for non-fractured stimuli. Potentially, the difference in agreement rate between Fractured and Non-fractured stimuli could be explained by the main effect on RT for Fractured. Responses to non-Fractured where consistently found to be slower than responses to Fractured stimuli. Following the Evidence Accumulation logic, this could have resulted in a lower accumulation rate for Agree decisions relative to Disagree decisions. In combination with a higher decision threshold in the AI advice blocks, this could have resulted in a lower proportion of agree decisions in the AI blocks, for the non-fractured stimuli. The specific experimental design we adopted here does not allow us to formally test this possibility though, given that we then would have to split the data into too many separate bins (Boag et al., 2024).

In Experiment 1, we did not observe a difference in agreement rate. We believe that the difference between the experiments may be related to differences in the expertise that was attributed to the human advisors (other participants vs. radiologists, cf. Hou & Jung, 2021). If the decision threshold for the AI advice (in both experiments) was more similar to that of the other participants than to that of the radiologists, then a difference in agreement rate in Experiment 1 may not have reached statistical significance in a traditional analysis.

## General discussion

Across two experiments, we observe a response time difference when participants are presented with AI advice versus human advice. Generally, AI advice is evaluated slower. At the same time, participants tend to agree less with AI advice on trials that were difficult (Experiment 2 only). These findings support that participants in our experiments display algorithm aversion (Dietvorst et al., 2015). We offer first insights in the mechanisms underlying algorithm aversion by fitting EAMs to the data of both experiments. Across experiments, we found that the threshold to agree or disagree with the AI advice was higher than the threshold to agree or disagree with human advice. Higher threshold estimates are often found under conditions where participants are asked to be cautious or avoid mistakes (Katsimpokis et al., 2020; Van Maanen et al., 2011, 2016). Thus, we explain the observed algorithm aversion by suggesting that participants are more cautious when advice comes from an algorithm.

Cautiousness in decision-making is a common phenomenon. Besides the aforementioned findings of more cautious decisions when instructed to do so (Katsimpokis et al., 2020; Van Maanen et al., 2011, 2016), cautiousness is also observed when there is increased uncertainty about the difficulty of the choice (Banca et al., 2015), when the potential gain or loss is larger (Dutilh & Rieskamp, 2016), or when participants are older (e.g., Archambeau et al., 2020; Starns & Ratcliff, 2012). These patterns seem to consistently suggest that when individuals aim to avoid mistakes, they strategically adjust their response threshold. This suggests that when presented with AI advice, individuals also aim to avoid mistakes. A possible reason for such behavior could be that individuals believe that the accuracy of AI advice is more uncertain than the accuracy of human AI. Another possible reason is that participants perceive the potential gain of following AI advice correctly as larger than the gain of following human advice. Such counterintuitive behavior could follow from the idea that the AI advice is more valuable than human advice, and therefore failing to follow advice is costlier. However, such explanations of the mechanism behind algorithm aversion observed in our experiments require further elaborate experimentation.

It is important to realize that our finding that algorithm aversion is due to an increase in response caution is not a foregone conclusion. An alternative mechanistic explanation of the different responses to AI and human advice could have been that algorithm aversion can be explained in terms of a larger starting point difference between the Agree and Disagree accumulators. That is, participants could require a differential amount of evidence to agree or disagree, and this differs between human and AI advice. While we indeed observe that the evidence required to agree is less than the evidence required to disagree, we did not observe any indication that this is different between human versus AI advice. A second alternative account is that algorithm aversion is expressed by lower accumulation rates in the AI advice blocks. This entails that participants rate the evidence to a lesser degree, for example, because they perceive information from AI advice as less certain. However, we found no evidence for differences in accumulation rate that would support such an account. Using formal models of cognition allows one to distinguish between these different ways in which algorithm aversion could be explained.

While we did find that participants set a higher threshold for algorithmic advice, the threshold distance also varied as function of the choice (to agree or disagree), which we interpreted as a prior preference to agree with advice rather than disagree. This effect, however, is between 5 and 30 times larger than the algorithm aversion effect—which is relatively small—, depending on the congruency condition and the experiment. On congruent trials, a small effect is expected because participants are quick to evaluate the congruent evidence, leading to a ceiling effect in terms of accuracy. High accuracy hampers the identification of differences in threshold parameters (Boag et al., 2024; Lerche et al., 2017) in general.

A limitation of our study is that we did not assess whether participants believed the framing of the experiment, with respect to whether advice really stemmed from AI, or whether it stemmed from humans as described (e.g., from fellow participants; see Experiment 1). Consequently, we cannot be certain that all participants performed the experiment as we intended. However, a set of similar studies that did ask post post-hoc about believability found that up to 95% of participants believed the cover stories (Özüdoğru et al., submitted). These numbers suggest that, also in our experiment, the majority of participants believed the framing.

To conclude, our study offers first insights into the cognitive mechanisms behind AI-supported decision-making. Across two experiments, participants evaluated medical advice from humans or AI. Using an established decision-making model, we found that individuals were slightly more hesitant to trust AI-generated advice than human advice. This result aligns with previous research on algorithm aversion (Dietvorst et al., 2015; Liefooghe, Oliveira, et al., 2023) but refines our understanding by pinpointing the cognitive mechanisms that express that aversion. The small effect sizes warrant replication, yet their consistency across experiments suggests they are meaningful. Although the immediate practical impact may seem minimal, even a slight shift in decision thresholds could lead to significant consequences over the many medical decisions made on a day-to-day basis.

## Supplemental Material

sj-docx-1-qjp-10.1177_17470218251398419 – Supplemental material for Modeling the cognitive processes of accepting clinical decision support Supplemental material, sj-docx-1-qjp-10.1177_17470218251398419 for Modeling the cognitive processes of accepting clinical decision support by Leendert van Maanen, Dominik Bachmann, Talha Özüdoğru, Macy Bouwhuizen and Baptist Liefooghe in Quarterly Journal of Experimental Psychology

## Notes

1. Specifically, on each trial, a warning time was drawn from an exponential distribution with rate 0.25. This corresponds to a mean warning time at 4 s, as a pilot study had indicated that most RTs (in a similar task) are faster than 4 s.
