# Supplementary material for: Modeling the cognitive processes of accepting clinical decision support
Source: Q J Exp Psychol (Hove). 2025 Nov 7;79(7):1662–73. doi: 10.1177/17470218251398419 (PMC13310272; doi:10.1177/17470218251398419)
Supplement: sj-docx-1-qjp-10.1177_17470218251398419 – Supplemental material for Modeling the cognitive processes of accepting clinical decision support [file sj-docx-1-qjp-10.1177_17470218251398419.docx]

Supplementary Material for:

**Modeling the cognitive processes of accepting clinical decision support**

Leendert van Maanen^1^, Dominik Bachmann^1,2^, Talha

Özüdoğru^1^, Macy Bouwhuizen^1^, Baptist Liefooghe^3^

^1^ Experimental Psychology & Helmholtz Institute, Utrecht University

^2^ Institute for Logic, Language, and Computation, University of Amsterdam

^3^ Social Health & Organizational Psychology, Utrecht University

# Supplementary Material A


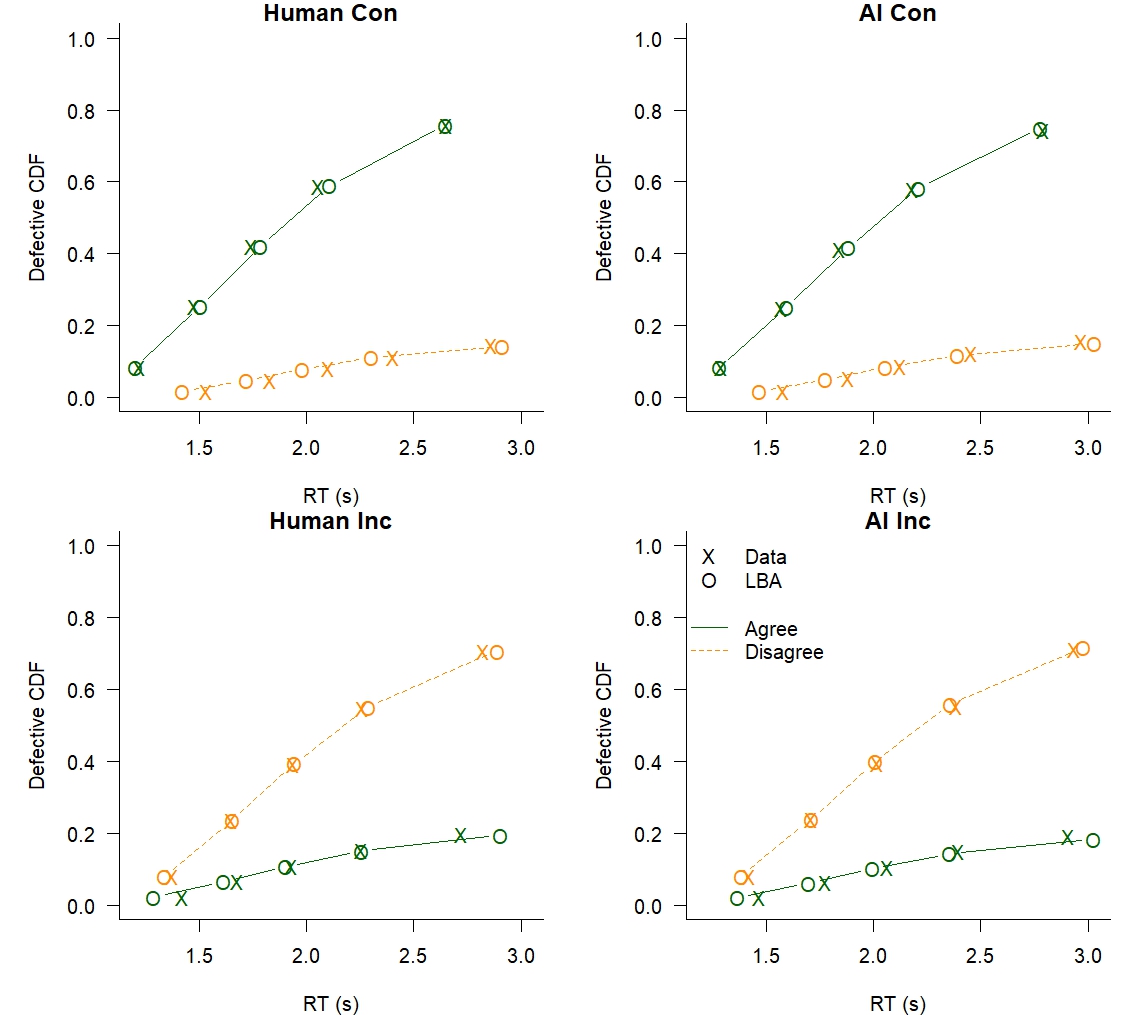
*Figure A. Fit of Model 2a (the optimal model) to the data of Experiment 1. The figure shows the defective cumulative density function (CDF), which represents the quantile probabilities for each RT quantile, scaled by the probability of the associated response (i.e., either Agree or Disagree). Shown are the Vincentized [0.1, 0.3, 0.5, 0.7, 0.9] quantiles, for both the data (X) and the model predictions (O), averaged across participants. Con: Congruent condition; Inc: Incongruent condition; LBA: Linear Ballistic Accumulator model.*

# Supplementary Material B


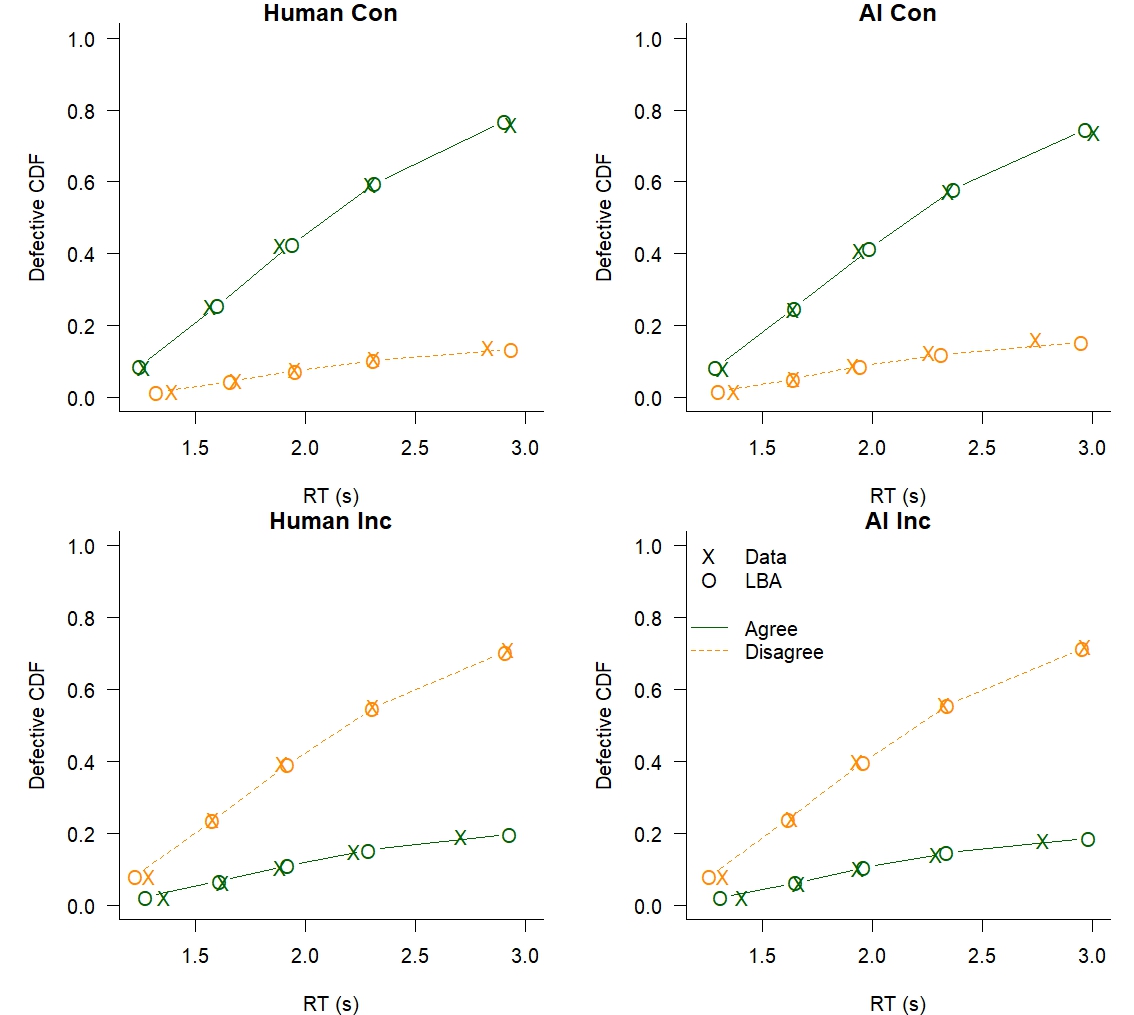


*Figure B. Fit of Model 1a (the optimal model) to the data of Experiment 2. The figure shows the defective cumulative density function (CDF), which represents the quantile probabilities for each RT quantile, scaled by the probability of the associated response (i.e., either Agree or Disagree). Shown are the Vincentized [0.1, 0.3, 0.5, 0.7, 0.9] quantiles, for both the data (X) and the model predictions (O), averaged across participants. Con: Congruent condition; Inc: Incongruent condition; LBA: Linear Ballistic Accumulator model.*
